# Niosomes as Vesicular Nanocarriers in Cosmetics: Characterisation, Development and Efficacy

**DOI:** 10.3390/pharmaceutics17030287

**Published:** 2025-02-21

**Authors:** Marko Lens

**Affiliations:** Leeds Institute of Medical Research, University of Leeds, Leeds LS9 7TF, UK; markolens@aol.com

**Keywords:** niosomes, delivery, transdermal, skin, cosmetics

## Abstract

In an era of significant developments in cosmetic chemistry and growing demand for efficacious skincare products, the efficient delivery of active molecules has been a challenge in formulations of cosmetics. In order to improve the performance of active compounds, the use of different nanotechnology-based systems have been explored in cosmetic chemistry. Niosomes, self-assembled vesicular nanocarriers, have been used in the cosmetic industry since the 1970s. The aim of this review is to provide a comprehensive overview of recent advancements in the encapsulation of active cosmetic compounds using niosomes as potential carriers for their sustained and targeted delivery. The review discusses the physicochemical, pharmacokinetic and pharmacodynamic properties of niosomes, including preparation methods, advantages and limitations. Various applications of niosomes in the cosmetic industry are presented together with the permeation and efficacy data from conducted in vitro and in vivo studies. Future perspectives of these nanocarriers for cosmetic applications are highlighted.

## 1. Introduction

The stratum corneum, the outermost layer of the epidermis, serves as a strong barrier that prevents active molecules from entering or passing through the skin. While small molecules can pass through the skin, larger molecules (molecular weight < 500 Da) are not able to cross the stratum corneum [1]. Active compounds can be transported across the stratum corneum via transepidermal (intercellular or intracellular) and transappendageal routes [2]. However, incapacity of active molecules to permeate the skin represents a major limitation for the performance of cosmetic formulations.

In the last few decades, significant research has been conducted in the field of cosmetic chemistry with the goal of creating and refining different transdermal delivery systems to enhance skin penetration and offer targeted and sustained delivery of active ingredients used in cosmetic formulations [3]. These advancements coincided with a growing demand for efficacious skincare products.

One of the most popular delivery systems in the cosmetic industry are niosomes, which were first created and patented by the French cosmetics giant L’Oréal. Nowadays, niosomes are frequently used in cosmetics and in other industries, such as the food and pharmaceutical industries [4].

This review provides an overview of niosomes as possible vehicles for the targeted and sustained delivery of active cosmetic ingredients and recent developments in the application of niosomes in cosmetic chemistry. The main physicochemical properties of niosomes and methods of their manufacturing as well as the evidence of niosome efficacy obtained in in-vitro and in-vivo research are discussed.

Niosomes: Composition

**Figure 1:**
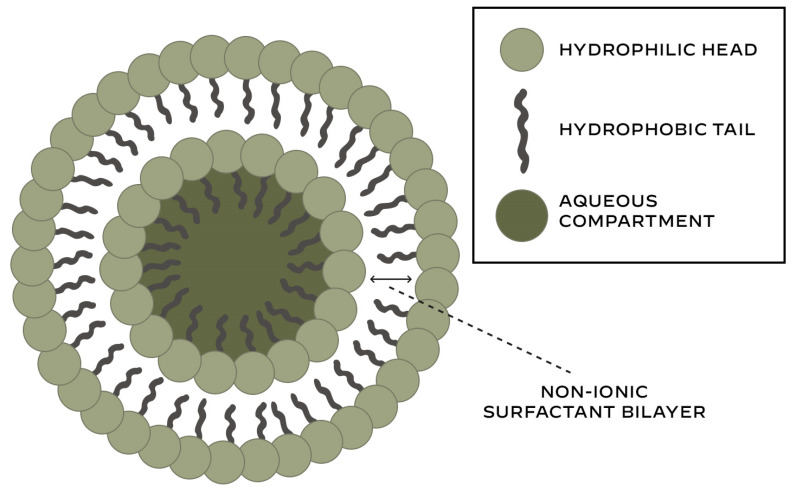
Structure of niosomes.

Niosomes are vesicular structures composed primarily of non-ionic surfactants with the addition of cholesterol and charge-inducing agents [5]. Niosomes are formed by self-clustering of non-ionic surfactants in aqueous media [6]. Thus, niosomes have a bilayer membrane surrounding an aqueous core (Figure 1).

The ability of non-ionic surfactants to self-assemble in an aqueous environment depends on: the hydrophilic–lipophilic balance (HLB), the critical packing parameter (CPP), the chemical structure and the phase transition temperature [7].

Due to their structure, niosomes can load both hydrophilic and lipophilic active compounds in the interior aqueous compartment and outer lipid layer, respectively [8]. The most often utilised non-ionic surfactant classes for niosome production (alkyl ethers, alkyl esters, alkyl amides and fatty acid esters, polymeric surfactants) are presented in Table 1.

Cholesterol is added to niosomes as a membrane stabiliser to enhance vesicle stability, decrease membrane permeability and boost niosome entrapment efficiency [9]. Positively or negatively charged molecules are added to bilayer membranes in order to increase the surface charge density and thus improve niosome vesicle stability and prevent aggregation [10].

Although surfactants are known as membrane destabilisers, non-ionic surfactants are able to form stable niosomal formulations due to their properties (excellent solubilisers, wetting agents and permeability enhancers with the ability to maintain pH up to physiological values). The most critical parameters in the selection of surfactants for niosome preparation are the hydrophilic–lipophilic balance (HLB) and critical packing parameter (CPP) [7]. However, the stability of niosomes is highly dependent on the lipid components and their degradation through hydrolysis and peroxidation.

**Scheme 1:**
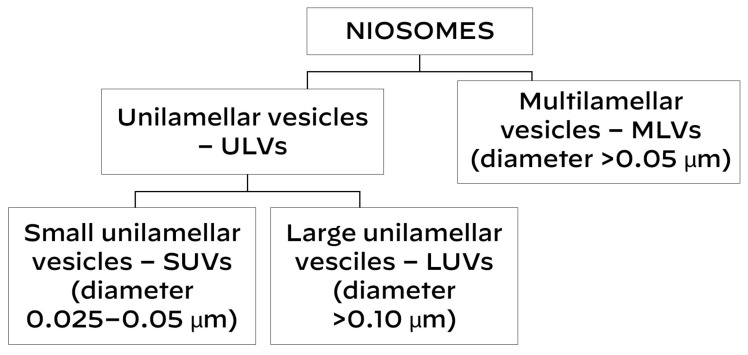
Classification of niosomes.

According to their size and quantity of membrane bilayers, niosomes are divided into various groups (Scheme 1):

1. Unilamellar vesicles.

They contain a single lipid bilayer. They can be:

(1) Small unilamellar vesicles (SUVs)—0.025–0.05 μm in diameter.
(2) Large unilamellar vesicles (LUVs)—≥0.10 μm in diameter.

2. Multilamellar vesicles (MLVs)

They contain multiple lipid bilayers; these niosomes have a diameter ≥ 0.05 μm [11].

## 2. Niosomes: Characterisation

The performance of niosomes is highly dependent on their main physicochemical characteristics: particle size (PS), polydispersity index (PDI), particle shape, surface morphology, zeta potential (ZP), entrapment efficiency, stability, in vitro drug release and in vivo performance.

The particle size indicates the capacity of niosomes to cross biological barriers like the blood–brain barrier, whereas the polydispersity index measures the distribution of niosome size in the formulation [12]. The particle size and polydispersity index are measured using dynamic light scattering (DLS) and photon correlation spectroscopy, while particle size and surface morphology are determined using electron microscopy, such as scanning electron microscopy (SEM) or transmission electron microscopy (TEM) [13].

Zeta potential refers to the electric potential at the slipping plane of a niosome vesicle. As an indirect measure of surface charge, the zeta potential predicts the stability of a nanoformulation [14]. Due to considerable electrostatic repulsion, formulations with a zeta potential that is either highly positive or negative (>±30 mV) are generally regarded as stable [15].

Entrapment efficiency (EE) is the percentage of an active compound that is successfully encapsulated relative to the total amount of active compound added during niosome formulation. The encapsulation of an active compound determines the pharmacokinetic and pharmacodynamic characteristics of niosomal nanocarriers [16]. Thus, the determination of the loaded and free active compound remains important, and it should be assessed after niosomes formulation using UV spectroscopy and high-performance liquid chromatography (HPLC) [17,18].

In vitro drug release represents an essential metric for the evaluation of the quality, safety and effectiveness of nanocarriers [19]. In vitro drug release kinetics help in the prediction of the in vivo performance of niosomes.

The stability of niosomes should be assessed at several time points during their storage at various temperatures. When assessing niosomes across time, three types of stabilities should be considered: physical, chemical and biological [20]. While physical stability refers to preservation of the structure, size and morphology of vesicles, chemical stability involves maintaining the bilayer composition of vesicles (surfactants, lipids and loaded active substance). The maintenance of niosome characteristics and biocompatibility in biological systems, which is critical to their efficacy and safety, is referred to as biological stability.

Niosomes have several advantages and few limitations [5,21,22]. Table 2 lists the main advantages and disadvantages of niosomes.

Together with liposomes, niosomes are the most often utilised vesicular delivery systems in cosmetics. Despite having structural similarities to liposomes, niosomes differ from them in a number of features [13,14,15,16,17,18,19,20,21,22,23,24,25]. The main differences between liposomes and niosomes are presented in Table 3.

## 3. Niosomes: Preparation Methods

Several methods have been developed to prepare niosomes. The selection of preparation method for niosome production is important as it affects their size, polydispersity index, stability and entrapment efficiency [26,27].

**Scheme 2:**
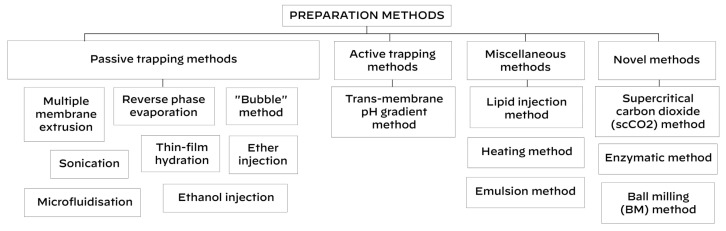
Methods for niosome preparation.

All methods for niosome synthesis can be classified in 4 groups (Scheme 2):

(1) Passive trapping methods (incorporation of active compounds into niosomes during their development).
(2) Active trapping method (incorporation of active compounds into niosomes after their synthesis).
(3) Miscellaneous methods.
(4) Novel methods.

Thin-film hydration, sonication, multiple membrane extrusion, reverse phase evaporation, microfluidisation, ether injection, the “bubble” method and ethanol injection are examples of passive trapping techniques, whereas the trans-membrane pH gradient method is an active trapping technique. Miscellaneous methods are: the lipid injection method, heating method and emulsion method.

### 3.1. Thin-Film Hydration Method

The thin-film hydration method, also referred to as the hand-shaking method, is a simple technique that is widely employed to create multilamellar niosomes [28].

**Figure 2:**
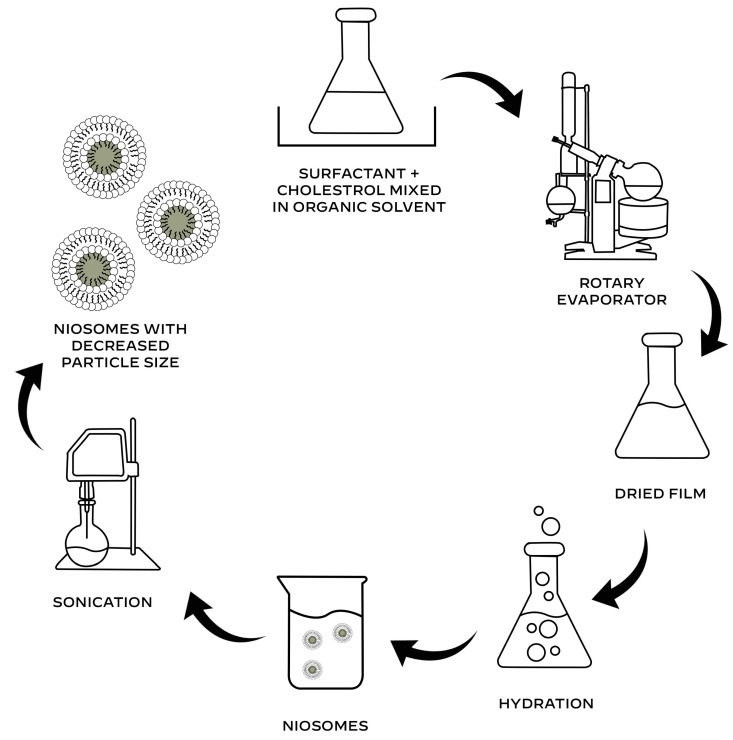
Thin-film hydration method for the preparation of niosomes.

This method involves dissolving the cholesterol and surfactant mixture in a volatile organic solvent. A rotary evaporator is then used to extract the solvent at room temperature until a thin layer of solid mixture is created. Niosomes that entrap the active compound are created when this dry film is rehydrated with the aqueous phase that contains the active component of interest at a temperature higher than the surfactant’s transition temperature [29,30,31,32]. The particle size of niosomes can be decreased by sonication (Figure 2). Multilamellar vesicles (MLVs) are created by a harsh hydration, whereas gigantic unilamellar vesicles (GULVs) are formed by gentle hydration [12].

The thin-film hydration technique and other methods with an aqueous phase can be used for the preparation of niosomes encapsulating hydrophobic active compounds. However, particular attention should be paid to the choice of media used for the preparation of niosomes entrapping active compounds that are poorly soluble in water.

### 3.2. Sonication Method

Sonication is a quick, easy, cost-effective and eco-friendly method for the production of small niosomes [33]. This process involves mixing the active ingredient with water (aqueous phase) and adding it to a lipid mixture of cholesterol and surfactant, which is subsequently sonicated [34].

### 3.3. Multiple Membrane Extrusion Method

A thin film is created by utilising a rotary evaporator to evaporate a solvent from a previously prepared mixture of surfactant, cholesterol and diacetyl phosphate. To produce uniformly sized niosomes, the film is hydrated with an aqueous solution that contains an active compound to create the suspension, which is then extruded through polycarbonate membranes arranged in series for up to eight passages [11,35].

### 3.4. Reverse Phase Evaporation Method (REV)

This method involves dissolving cholesterol and a surfactant in an organic solution. After adding an aqueous phase that contains the active compound, the two immiscible phases that arise are homogenised and subjected to sonication [36,37,38]. The organic solvent is then eliminated by rotational evaporation of the resulting emulsion. Consequently, the final niosomal suspension is produced.

### 3.5. Microfluidisation Method

The submerged jet principle is the foundation of the microfluidisation technique. In the interaction chamber, two fluidised streams (one containing a surfactant and the other an active compound) interact and merge at extremely high velocity in carefully defined microchannels, resulting in the self-assembly of niosomes [39,40,41]. The main advantages of this technique include the production of smaller niosomes with excellent homogeneity and reproducibility and the elimination of the need for the use of organic solvents [29].

### 3.6. Ether Injection Method

This method involves dissolving cholesterol and a non-ionic surfactant in ether, then gradually injecting the mixture through a needle into an aqueous phase that contains an active ingredient that is maintained at a temperature higher than the boiling point of the organic solvent [42,43]. Gradual vaporisation of ether generates unilamellar niosomes, which have a diameter of 50–1000 nm [44].

### 3.7. The “Bubble” Method

In this one-step procedure, a non-ionic surfactant and cholesterol are mixed in a buffer and put in a three-neck round-bottom flask, also known as a “bubbling unit”, that is set in a water bath [8,45]. While the nitrogen is supplied through the third neck, the temperature is controlled by a thermometer in the second neck and a water-cooled reflux in the first neck. Niosomes are created by bubbling the dispersion with nitrogen gas at 70 °C after it has been mixed for 15 s with a high-shear homogeniser. This technique does not require the use of organic solvents.

### 3.8. Ethanol Injection Method

Non-ionic surfactants and cholesterol are dissolved in ethanol. This lipid ethanol solution is injected in a stirred aqueous solution containing an active compound. A rotary evaporator is used to remove the organic phase [46,47,48]. The size of the created nanovesicles is reduced using an ultrasonic probe. One of the main problems associated with this method is an amount of residual ethanol.

### 3.9. Trans-Membrane pH Gradient Method

In this method, non-ionic surfactant and cholesterol are dissolved in chloroform [49]. To create a thin layer on the flask wall, the solvent is subsequently eliminated using a rotary evaporator operating at lower pressure. Next, using a vortex mixer, this film is hydrated with citric acid at a pH of 4.0. Following three cycles of freezing and thawing, formed multilamellar vesicles are sonicated [11,44,50]. After adding and vortexing an aqueous solution containing an active compound, the pH of the mixture is increased to 7.0–7.2 using 1M disodium phosphate. Multilamellar niosomes are then created by heating this mixture for ten minutes at 60 °C. This niosome manufacturing technique produces multilamellar vesicles with high stability and encapsulation efficiency [51].

### 3.10. Lipid Injection Method

This environmentally friendly method does not require an organic solvent. Niosomes are created by quickly injecting a melted combination of cholesterol and non-ionic surfactant into a heated, highly agitated aqueous phase that contains an active compound [5,11,52].

### 3.11. Heating Method

The heating method was initially developed by Mozafari et al. to produce liposomes [53]. Nowadays, this process involves hydrating surfactants in a pH 7.4 phosphate buffer for one hour at room temperature. Meanwhile, an active compound and glycerol (final concentration 3%, *v*/*v*) are added to the cholesterol dispersion and stirred for fifteen minutes at 120 °C (final concentration 3%, *v*/*v*) [54]. To create niosome vesicles, samples are combined and heated while being stirred (800–1000 rpm) for 30 min in a nitrogen environment [55].

### 3.12. Emulsion Method

In this simple method, niosomes are formed by preparing oil in water (o/w) emulsion by adding an organic solution of surfactant and cholesterol to the aqueous solution with an active compound [44]. Niosomes are dispersed in the aqueous phase after the organic solvent evaporates [11].

### 3.13. Novel Methods

In recent years, a number of innovative methods have been developed to prepare nanovesicular carriers. These techniques include the enzymatic method, supercritical carbon dioxide (scCO_2_) method and the novel ball milling (BM) method [11,46,56].

In 2008, Manosroi et al. introduced the supercritical carbon dioxide (scCO_2_) method for niosome preparation [56]. This one-step procedure involves mixing an active ingredient in a glass cell with surfactant (Tween 61) and cholesterol in a 1:1 ratio. The CO_2_ gas is then injected into the cell while the temperature and pressure are kept at 60 °C and 200 bar, respectively. After 30 min of stirring all ingredients with a magnetic stirrer, the pressure is reduced, and niosomal dispersion with big unilamellar niosomes ranging in size from 100 to 440 nm are formed. With this method, niosomes can be prepared with or without ethanol: niosomes prepared by the scCO_2_ method with ethanol have a higher entrapping efficiency than those prepared without ethanol. The scCO_2_ method is considered environmentally friendly as it does not require the use of toxic and flammable solvents.

## 4. Application of Niosomes as Carriers of Active Cosmetic Compounds

Recent advancements in the field of cosmetic chemistry and technology have resulted in the preparation of numerous nanovesicular carrier systems encapsuling different active cosmetic molecules. Various types of niosomes have been prepared by entrapping phytochemicals, anti-oxidants, hyaluronic acid, vitamins, peptides, sunscreens and many other active molecules, with the efficacy suitable for cosmetic use.

### 4.1. Encapsulation of Plant Extracts in Niosomes

Because of its moisturising, anti-inflammatory, and anti-oxidant qualities, rice bran (*Oryza sativa*) has been used in cosmetics for a long time. Using the supercritical carbon dioxide approach, Manosroi et al. encapsulated rice bran extract containing ferulic acid, gamma-oryzanol and phytic acid into niosomes [57]. The entrapment efficiencies were 64.5% for ferulic acid, 47.5% for gamma-oryzanol, and 54.9% for phytic acid. These niosomes were considered suitable for topical administration despite their slightly larger size (480.9 ± 270.8 nm) and PDI of 1.5 [58,59]. An in vivo study including 30 volunteers who used topical gel and cream formulated with rice bran bioactive compounds entrapped in niosomes for 28 days showed significant improvements in skin thickness, elasticity, roughness and moisture. These results suggests that nanocarriers with rice bran can be useful in cosmetic formulations with anti-ageing claims.

The same team prepared niosomes loaded with different percentages of unsaturated fatty acids containing semi-purified fraction no. 3 of rice bran extract (OSF3) [60]. Niosomes containing 0.5% OSF3 had a size of 12–220 nm and encapsulation efficiency of 86.2%. The use of niosomes loaded with OSF3 has been proposed for anti-hair loss products because of their 5α-reductase inhibitory action. Results from the study of the trans-follicular delivery of the gel containing OSF3-loaded niosomes performed on porcine skin suggested that these niosomes are suitable for topical treatment of androgenic alopecia [61].

Later, Manosroi et al. used the thin-film hydration method to create niosomes encapsulating purple glutinous rice extract rich in flavonoids and anthocyanins [62]. These niosomes had a low PDI of 0.337 and a size of 135.9 ± 4.56 nm as a result of sonication. The HPLC analysis revealed that although the amount of anthocyanin in the purple glutinous rice extract was 0.35 µg/1 mg of the extract, the formation of niosomes reduced the amount of anthocyanin to 0.19 µg/1 mg of the extract (approximately 45% reduction) because of the heat-induced hydrolysis of anthocyanin. Results from an in vivo study conducted on 20 volunteers demonstrated that after 28 days of use, the cream with niosomes loaded with 1% w/v of the purple glutinous rice extract significantly decreased skin hyperpigmentation while improving skin elasticity and hydration. The achieved results indicate that nanocarriers with the purple glutinous rice extract can be an efficacious active ingredient in anti-ageing cosmetic formulations.

Ginger (*Zingiber officinale*) root extract has been widely utilised in cosmetic formulations due to its strong anti-inflammatory, anti-oxidant and antimicrobial properties [63]. Using a thin-film hydration technique, Abdallah et al. created niosomal vesicles filled with ginger extract [64]. These niosomes were around 230 nm in size, with a PDI of 0.31, and exhibited an entrapment efficiency of 63.2%. They added ginger-loaded niosomes in a sesame oil-based niosomal emulgel which in an ex vivo study demonstrated to be significantly better transdermal permeability when compared with gel, emulgel and a suspension of ginger extract (J_ss_ values of 81.84 µg/cm^2^.h, 62.59 µg/cm^2^.h, 51.24 µg/cm^2^.h and 30.95 µg/cm^2^.h, respectively). Ginger extract niosomal emulgel also showed improved anti-inflammatory activity. The obtained results suggested that the combination of niosomes and emulgel (incorporation of niosomes in emulgel) enhances both permeation and the anti-inflammatory effect of the active compound loaded in niosomes [65].

Ongtanasup et al. used the thin-film hydration technique and sonication to create niosomes encapsulated with ginger root extract [66]. They created a number of formulations with varying proportions of cholesterol and non-ionic surfactants (Span 60 and Tween 80) in order to maximise the drug-loading effectiveness of ginger extract and prolong its in vitro anti-inflammatory action. According to various experiments, the optimised niosomal formulation containing the highest amount of Tween 80, the lowest amount of Span 60 and a moderate cholesterol amount (20: 12.5, 5 w/w) had a particle size of around 265 nm, polydispersity index of 0.26 and encapsulation efficiency of 54.71%.

Numerous studies have documented that the main components of a medicinal plant gotu kola (*Centella asiatica*), asiaticoside and madecassoside exhibit a broad range of pharmacological activities [67]. Gotu kola has been used to treat a variety of dermatological conditions as well as an important active ingredient in cosmetic formulations [68]. To improve the permeability of the extract of gotu kola, Wichayapreechar et al. used the thin-film hydration technique to entrap it into niosomes. This resulted in the formation of vesicles with a particle size of 155 nm, a drug loading capacity of up to 7%, and an encapsulation efficiency of up to 77% [69]. To increase the transdermal permeability of these niosomes, Wichayapreechar et al. added hyaluronic acid as a copolymer to create surface-modified niosomes, which had larger size, lower drug loading capacity and unchanged encapsulation efficiency. Tests on animal skin revealed that gotu kola extract-modified niosomes had improved asiaticoside penetration through the stratum corneum and enhanced dermal retention, indicating that these nanocarriers may be useful for topical formulations.

Sugar apple (*Annona squamosa*) leave extract (ASLE) exhibits strong anti-oxidant, anti-inflammatory and antimicrobial activities due to its high content of phenolic compounds and flavonoids [70]. Using the thin-film hydration approach, Mohamad et al. entrapped sugar apple leaf extract into niosomes [71]. The size of the prepared niosomes was approximately 100 nm, and their entrapment efficiency was 90%. Studies conducted on animal skin samples showed that ASLE-loaded niosomes offered noticeably superior protection against UVA damage compared with native plant extract.

Naturally derived enzymes have been used in the preparation of various cosmetic products. One of the most commonly utilised enzymes in exfoliating and skin-resurfacing cosmetic formulations is papain, a proteolytic enzyme isolated from the latex of papayas (*Carica papaya*) [72]. Manosroi et al. prepared a gel with papain-loaded niosomes (size 220–520 nm), reporting improved chemical stability and transdermal absorption of this gel compared with a gel with free papain [73]. A histological examination conducted on animal models demonstrated that papain-loaded niosomes reduce skin scarring, suggesting that these nanocarriers can provide benefits when used in topical formulations for the treatment of scars.

### 4.2. Encapsulation of Anti-Oxidants in Niosomes

Targeting oxidative stress has been the primary objective of many cosmetic formulations in recent years. As a result, we saw a notable rise in the use of flavonoids and polyphenols, which are naturally occurring anti-oxidants that scavenge free radicals, unstable molecules responsible for the oxidative skin damage.

Because of its superior anti-oxidant and anti-inflammatory properties, a naturally occurring phenolic compound resveratrol has drawn a lot of interest in the preparation of numerous topical medicinal and cosmetic formulations [74]. In order to overcome its low transdermal absorption, Pando et al. encapsulated *trans*-resveratrol into niosomes prepared using two different methods (a thin-film hydration method followed by a sonication and an ethanol injection method) [75]. Two unsaturated fatty acids, oleic and linoleic acids, were added as penetration enhancers. Ex vivo tests conducted on animal models showed that niosomes prepared by the ethanol injection method had smaller size, higher entrapment efficiency, better stability and superior skin penetration in comparison with niosomes formed using the thin hydration method.

Tavano et al. prepared niosomes entrapped with resveratrol, showing controlled and enhanced percutaneous permeation of these vesicles compared with free resveratrol solution [76]. Additionally, they observed that the ability of niosomes to scavenge free radicals is improved when resveratrol is entrapped together with alfa-tocopherol and curcumin, which has been explained by their synergistic anti-oxidant activity.

Curcumin, a major polyphenol of turmeric (*Curcuma longa*), exhibits multiple potential skin benefits primarily through its anti-oxidant, anti-inflammatory and antimicrobial mechanisms [77]. However, the use of curcumin in cosmetic formulations is limited *due to the lack of its solubility* in aqueous solvent, poor stability and low bioavailability [78]. Kolahdooz et al. encapsulated curcumin in niosomes produced by the thin-film hydration method (diameter 13 ± 2.20 nm and PDI of 0.2), achieving an encapsulation efficiency of 86% [79]. A pilot randomised controlled trial evaluating the efficacy of these niosomes added to hyaluronic acid and a marine collagen gel-based formulation showed that topically applied curcumin-loaded niosomes have significant therapeutic effects on skin lesions and the healing process in patients with psoriasis. These findings imply that curcumin-niosomes may be beneficial in cosmetic formulations designed to reduce skin redness and irritation.

In order to increase curcumin’s efficiency and skin delivery, Sadeghi Ghadi et al. co-encapsulated curcumin and quercetin in niosomes using the thin-film hydration method with the addition of hyaluronan polymeric solution. The entrapment efficiency of curcumin and quercetin was 98.8% and 93.1%, respectively [80]. Niosomes with curcumin and quercetin appear to be efficacious active ingredients for cosmetic formulations targeting oxidative stress and damage of the skin.

Ascorbic acid, commonly known as vitamin C, is a widely used anti-oxidant ingredient in cosmetic products. Unfortunately, poor stability, easy degradation in oxidation pathways and low skin penetration of pure ascorbic acid represent the major obstacles for its use [81]. To increase the stability and bioavailability of ascorbic acid, several encapsulation techniques have been employed.

Shabani Dargah and Hadjizadeh encapsulated ascorbic acid in niosomes that were further coated with 10% w/w hyaluronic acid (HA), reaching an encapsulation efficiency of 56.5% [82]. High drug penetration and accumulation of ascorbic acid entrapped in HA-coated niosomes were found to be 116.5 and 134.8 µg/cm^2^, respectively. These results confirmed that encapsulation of ascorbic acid in niosomes led to the significant improvement in the performance of ascorbic acid in the skin.

To overcome stability and penetration issues of pure ascorbic acid, Kandil et al. explored the use of magnesium ascorbyl phosphate (MAP), a stable esterified derivative of ascorbic acid [83]. Using the thin-film hydration method, they prepared niosomes (approximately 138 nm in size) loaded with MAP with an encapsulation effectiveness of 86.8% and added them to carbol gel to reach a final MAP concentration of 5% w/w. Conducted experiments revealed that carbopol gel promotes the permeation and accumulation of MAP-loaded niosomes into the skin due to its water retention effect. Results from the clinical study including 40 individuals (mean age 41.9 years) showed that the application of MAP-niosomal gel significantly reduced the appearance of melasma and facial hyperpigmentation at the 6-month follow-up.

### 4.3. Encapsulation of Sunscreens in Niosomes

Despite the fact that the global revenue in the sun protection segment of the beauty and personal care market continues to rise, the development and application of sunscreens confronts a number of challenges [84]. Zinc oxide (ZnO) is one of the most commonly used sunscreens to protect the skin the skin against solar UV irradiation. ZnO nanoparticles (ZnO-NPs) produced by the green synthesis method have recently drawn interest due to their low toxicity and high biodegradability [85]. To enhance the biological potential of ZnO-NPs, Rezaei et al. loaded them into niosomes prepared using the thin-layer hydration method, reaching a final concentration of ZnO-NPs of 1 mg/mL [86]. Niosomes loaded with ZnO-NPs had a size of about 256 nm, PDI 0.136, high stability (zeta potential -23.7) and an encapsulation efficiency of 31.3%. According to their research, only 31.2% of ZnO-NPs were released from niosomes within the first 8 h, whereas 86.1% of free ZnO-NPs were released in the medium during the same period. These findings indicated that niosomes containing ZnO-NPs prolonged the protective activity of Zn-NPs acting as effective carriers for targeted delivery.

Mohamad et al. prepared a sunscreen phenylbenzimidazole-5-sulfonic acid (PBSA) into niosomes subsequently coated with chitosan and aloe vera to create a nanocarrier polymer with an entrapment efficiency of 80% [87]. The conducted experiments showed that coated niosomal nanostructures allow for controlled release of PBS and limited epidermal penetration due to enhanced deposition on the surface of the skin, allowing for extended UV protection.

The potential use of nanostructures in the protection against UV light has been explored by Cerqueira et al., who prepared unilamellar niosomes loaded with a mixture of three different sunscreens (octyl methoxycinnamate, phenylbenzimidazole sulfonic acid and diethylamino hydroxybenzoyl hexyl benzoate), achieving an entrapment efficiency of over 45% for each sunscreen [88]. In vitro and in vivo studies evaluating two different niosomal formulations demonstrated that the created nanostructures show improved retention in the skin, providing excellent photoprotective activity.

### 4.4. Niosomal Preparations in the Treatment of Dermatological Disorders

Although numerous niosomal preparations have been prepared for use in dermatological products to treat different skin disorders, this review did not address niosomes encapsulating active molecules classified as drugs but only those considered as cosmetic ingredients. However, some dermatological conditions may benefit from both medical and cosmetic treatments. Acne vulgaris, a widespread problem among young people, is a good example.

Benzoyl peroxide (BPO) is one of the most commonly utilised chemicals for acne treatment. While BPO is permitted for professional usage only in the EU at a maximum concentration of 0.7% in finished cosmetic products, it is licensed as an over-the-counter topical treatment for acne in the US. To enhance BPO permeation, Goyal et al. used the thin-film hydration technique to load BPO into niosomes (309 nm) with a 58% encapsulation efficiency and drug release of 72.9% after 24 h [89]. In vivo study after 4 days showed that BPO-loaded niosomal gel exhibited a stronger anti-inflammatory effect and higher reduction rate in the CFU count of *Propionobacterium acnes* (log CFU/mL) than plain BPO, with 54.2% versus 50.9% and 6.14 versus 5.46, respectively. However, in recent years, many consumers have raised some concerns regarding the safety of BPO in cosmetic formulations for acne treatment, looking for natural and botanical-derived alternatives.

Using the sonication process, Kanpipit et al. added 0.5% salicylic acid (SA) and 0.25% oleoresin from *Dipterocarpus alatus* to a niosomal formulation in order to increase the permeability of SA and extend its controlled release [90]. With their 216 nm size and 84.1% encapsulation efficiency, the niosomes decreased the expression of anti-inflammatory proteins and enhanced their inhibitory effects on *Propionobacterium acnes*, indicating that the SA-ODA niosome formulation may find use in the development of topical anti-acne products.

Budhiraja and Dhingra encapsulated rosmarinic acid (ROA) into niosomes (814 nm) with a 65% entrapment efficiency using the reverse phase evaporation process [91]. They added ROA-niosomes to carbopol gel, which demonstrated a 49.8% in vitro drug release after 24 h and a robust inhibitory effect against *Staphylococcus aureus* and *Propionobacterium acnes*. According to an in vivo study, the ROA-niosomal gel significantly inhibited inflammation compared with the gel containing simple ROA, with 58.2% and 40.9% inhibitions in 3 days, respectively. According to these findings, ROA-niosomes can be used in the treatment of acne and blemishes.

## 5. Conclusions

In the past few decades, significant efforts have been made to research and develop different technologies for the enhanced delivery and efficacy of active cosmetic molecules. The use of niosomes as vesicular nanocarriers has been explored in the field of cosmetic chemistry and skincare due to their excellent stability, ability to entrap both hydrophilic and lipophilic molecules and potential to improve the penetration of active ingredients. Despite being structurally similar to the commonly used liposomes, niosomes offer important advantages, including greater stability, lower cost and easy preparation and storage.

While there is no doubt that niosomes offer many advantages, we should not ignore some limitations and challenges in the manufacturing and application of niosomes in cosmetics. First of all, niosomes preparation can be complex, and the batches produced can differ greatly from one another. The type and concentration of surfactants used to prepare niosomes, temperature and pH are the main causes of uneven quality in niosome formulation. Particular attention should be paid to the selection of surfactants, particularly because the type and concentration of surfactants may induce a toxic effect of niosomes. The gel–liquid transition temperature of the surfactants used for niosomes preparation had an impact on the encapsulation efficiency and permeability and stability of niosomal formulation. However, the entrapment of active compounds into niosomes depends on various factors that not only affect the encapsulation process but also may cause alteration of the pharmacokinetics of the encapsulated compound. Lastly, the fact that niosomes may demonstrate notable variations in their in vitro and in vivo behaviour may have a strong impact on the practical application of niosomes.

Future studies and advancements in niosome-based delivery systems should address the challenges of niosome preparation and production scaling while maintaining the consistent quality and performance of niosomal formulations. Despite the fact that numerous studies have shown that niosomes may contain a variety of active chemicals to maximise their skin delivery and efficacy, some active compounds may not be suitable for niosomal delivery because of their limited solubility. Thus, to improve skin delivery and boost the efficacy of active ingredients used in cosmetic products, further research is needed. Researchers should be aware of the necessity of selecting appropriate surfactants for niosome preparation given the fact that the type of surfactant used is the primary factor determining the successful encapsulation of active compounds in niosomal vesicles.

Further research into the widespread use of niosome delivery systems in cosmetics is necessary because the cosmetic industry currently has limited understanding and acceptance of niosomes due to their comparatively limited research compared with other delivery systems like liposomes. Innovative techniques for niosome preparation, loading and modification can increase their commercial potential in cosmetic industry.

Because the cosmetic industry is expanding rapidly with a growing demand for high-performance skincare products, especially in the field of anti-ageing formulations, exploring the further potential of the current topical, dermal and transdermal delivery systems and introducing innovative systems for the targeted delivery of active cosmetic molecules is crucial in the formulation of efficacious and science-led cosmetics based on the latest research findings and cutting-edge technology. Given the growing global trend of sustainable, eco-friendly and personalised skincare concepts, niosome-based technology may have a bright future in the delivery of a variety of natural and bioactive compounds. However, the use of new niosome delivery systems may face regulatory scrutiny, requiring extensive safety and efficacy testing before they can be marketed.

## Figures and Tables

**Table 1:** Non-ionic surfactants used for niosome formation.

Types of Non-Ionic Surfactants	Examples
ALKYL ETHERS	Polyoxyethylene alkyl ethers (Brij—30, 35, 52, 58, 72, 76, 92) Alkyl glycerol ethers
ALKYL ESTERS	Polyoxyethylene sorbitan fatty acid esters (Tweens—20,40, 60, 65, 80, 85) Sorbitan fatty acid esters (Spans—20, 40, 60, 65, 80, 85)
ALKYL AMIDES	Alkyl galactosides (Octyl-decylpolyglucoside, Decylpolyglucoside) Alkyl glucosides (C-Glycoside derivative surfactants)
FATTY ACID ESTERS	Stearic acid Palmitic acid Myristic acid
FATTY ALCOHOLS	Stearyl alcohol Myristyl alcohol Acetyl alcohol
POLYMERIC SURFACTANTS	Poloxamer 188 (Pluronic F-68) Poloxamer 407 (Pluronic F-127)

**Table 2:** Main advantages and disadvantages of niosomes.

Advantages	Disadvantages
- Can load hydrophilic, lipophilic and amphiphilic molecules - Good chemical stability - Allow sustained/controlled release of active compound and targeted diffusion - Biocompatible, biodegradable and non-immunogenic - Long storage and no special handling and storage conditions - Improve stability of entrapped active compound - Long shelf life	- Low physical stability - High probability of aggregation - High risk of leakage or hydrolysis of entrapped active compound - May fuse together, leading to changes in membrane composition and properties - Problems with sterilisation - Time-consuming production - Requires special equipment for production

**Table 3:** Comparison between liposomes and niosomes.

Feature	Liposomes	Niosomes
Composition	Phospholipids	Non-ionic surfactants
Origin of components	Naturally occurring	Synthetic
Size	10–3000 nm	10–1000 nm
Chemical stability	Low	Very good
Storage conditions	Special conditions required	No special requirements
Production costs	More expensive	Less expensive
